# Observation of Interlayer Excitons in Mixed-Dimensional MoS_2_ and InGaN/GaN Quantum Well Heterojunctions

**DOI:** 10.1021/acsami.5c16066

**Published:** 2025-11-18

**Authors:** Do Wan Kim, Seokje Lee, Yongmin Baek, Kangmin Jeon, Jinwoo Song, Kwangsik Jeong, Seungjin Lee, Jae Woo Kim, Seokho Kim, Gi Wan Jeon, Ki Kang Kim, Gyu-Chul Yi, Dong Hyuk Park, Kyusang Lee

**Affiliations:** † Department of Electrical and Computer Engineering, 2358 University of Virginia, Charlottesville, Virginia 22904, United States; ‡ Department of Physics and Astronomy, Institute of Applied Physics, 26725 Seoul National University, Seoul 08826, Republic of Korea; § Research Laboratory of Electronics, Massachusetts Institute of Technology, Cambridge, Massachusetts 02139, United States; ∥ Department of Mechanical Engineering, Seoul National University, Seoul 08826, Republic of Korea; ⊥ Department of Chemical Engineering, Program in Biomedical Science and Engineering, 26718 Inha University, Incheon 22212, Republic of Korea; # Department of Physics, Yonsei University, Seoul, Gangwon-Do 26493, Republic of Korea; ∇ Department of Energy Science, 35017 Sungkyunkwan University (SKKU), Suwon 16419, Republic of Korea; ○ Center for Integrated Nanostructure Physics (CINAP), Sungkyunkwan University (SKKU), Suwon 16419, Republic of Korea; ◆ Particle Beam Research Division, 65405 Korea Atomic Energy Research Institute (KAERI), Gyeongju-Si, Gyeonbuk 38180, Republic of Korea; ¶ Department of Physics, Sungkyunkwan University (SKKU), Suwon 16419, Republic of Korea; $ Department of Materials Science and Engineering, University of Virginia, Charlottesville, Virginia 22904, United States

**Keywords:** interlayer excitons, heterojunction, InGaN quantum well, transition metal dichalcogenides, exciton dynamics, band alignment engineering

## Abstract

Mixed-dimensional heterojunctions (HJs) between compound semiconductors and transition metal dichalcogenides (TMDCs) provide a versatile platform for modulating interfacial exciton dynamics. While compound semiconductors offer precise compositional control for bandgap tuning across wide spectral ranges, they exhibit weak exciton binding energies that limit exciton stability at room temperature. Mixed-dimensional HJs address this limitation by integrating compound semiconductors with TMDCs, whose strong quantum confinement and reduced dielectric screening enable the formation of stable interlayer excitons with enhanced light-matter coupling. Here, we demonstrate a mixed-dimensional heterojunction comprising trilayer MoS_2_ interfaced with an Al_2_O_3_/InGaN/GaN single quantum well (QW), designed to investigate interlayer exciton behavior. Quantum confinement in the QW localizes carriers near the heterointerface, allowing direct observation of interlayer excitonic states. Low-temperature photoluminescence measurements revealed a distinct emission peak at 2.02 eV, indicating the formation of interlayer excitons at the heterointerface. This strategy offers a unique approach for engineering exciton dynamics in mixed-dimensional systems, with implications for optoelectronic devices that leverage tailored interfacial exciton dynamics.

## Graphical abstract

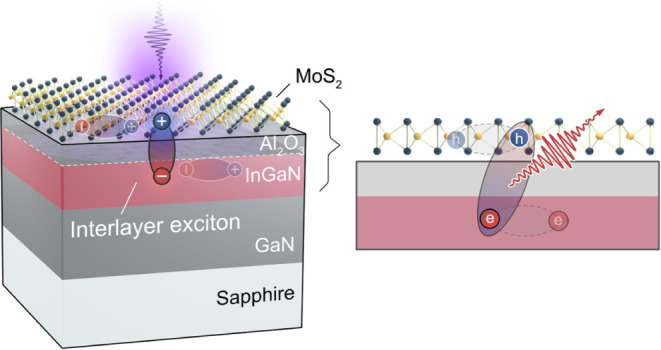

## Introduction

Carrier lifetime fundamentally governs the efficiency of photonic and optoelectronic devices, as photogenerated electrons and holes need to persist long enough to be transported and converted into light or electrical signal prior to recombination.^1,2^ In low-dielectric systems such as organic semiconductors and transition-metal dichalcogenides (TMDCs), Coulomb attraction binds electrons and holes into bound pairs known as excitons, and these excitonic states dominate carrier dynamics rather than free-carrier transport.^1,2^ Reduced dielectric screening in these materials results in large exciton binding energies far exceeding those in inorganic semiconductors, enabling excitonic states to remain stable even at room temperature. Although intrinsic material properties determine exciton lifetime within individual layers, heterostructures offer opportunities to engineer excitonic dynamics.

Spatial separation of electrons and holes across heterogeneous materials permits the formation of interlayer excitons with extended lifetimes, an attribute that has drawn substantial attention for efficient light-matter coupling in optoelectronic applications.^3–6^ The excitonic behavior at a heterointerface, however, sensitively depends on both band alignment and atomic registry.^7^ Precise control over layer thickness, composition, and material pairing enables tuning of exciton binding energy and lifetime, thereby directly influencing exciton-to-charge conversion and recombination dynamics.^8–11^

Despite the promise of exciton engineering in heterostructure, a fundamental challenge remains; Wannier–Mott excitons in 3D compound semiconductors exhibit relatively weak binding energies, making exciton stabilization at room temperature difficult.^11–13^ Thus, 2D-3D mixed-dimensional heterojunctions (2D/3D-HJs), formed by integrating TMDCs with quantum-structured compound semiconductors, offer a compelling solution to intrinsic material limitations. TMDCs provide strong quantum confinement and reduced dielectric screening, resulting in exciton binding energies on the order of several hundred meV,^14–16^ whereas compound semiconductors benefit from mature and well-established growth techniques capable of precise compositional control and sophisticated quantum structure design. This complementary combination enables engineered band alignment and enhanced interfacial coupling.

Recent approaches to heterostructures have shown that quantum well structures promote coupling of the carriers by spatially confining them within narrow potential wells. This spatial confinement enhances the binding energy of interlayer excitons by up to 16-fold compared to bulk counterparts through dielectric confinement effects.^17^ These quantum well structures also enable precise control of light–matter interactions by modulating sub-band energies and excitonic resonances.^18,19^ Among candidate materials, III–N semiconductors provide exceptional versatility with bandgaps ranging from 0.7 eV (InN) to 6.0 eV (AlN) through compositional tuning of indium (In), aluminum (Al), and gallium (Ga), thus offering a broad tunability window for QW design.^20–28^ Integrating TMDCs with III–N QWs therefore introduces new degrees of freedom in band alignment and lattice engineering, overcoming the weak interlayer coupling and band-offset mismatches that limit purely 2D or 3D material-based heterostructures. This integration enables the stabilization of interlayer excitons at room temperature, offering a promising route to demonstrate high-performance mixed-dimensional optoelectronic devices.

In this study, we demonstrate the formation of interlayer excitons in a mixed-dimensional heterojunction by engineering the energy level at the interface of multilayer MoS_2_ with an InGaN quantum well. Trilayer MoS_2_ was chosen for its strong photoresponsivity and greater optical absorption compared to monolayers, owing to its increased thickness and reduced sensitivity to substrate-induced effects such as strain.^16,29–31^ Meanwhile, the InGaN QW provides tunable excitonic resonance by controlling its thickness and composition.^16,18,19^ We introduced an ultrathin (2 nm) Al_2_O_3_ barrier between the MoS_2_ and InGaN layers to create a confined potential landscape and suppress exciton annihilation at the interface. This 2 nm-thick Al_2_O_3_ barrier enables photogenerated carrier coupling across the junction, thus promoting formation of interlayer excitons, while preserving the confinement integrity of the potential well. Low-temperature photoluminescence (PL) measurements of this mixed-dimensional HJ revealed an additional emission peak, attributed to radiative recombination of interlayer excitons. Notably, this feature disappears in the structure of InGaN/MoS_2_ without the Al_2_O_3_ spacer, indicating that an appropriately engineered energy barrier is essential for stabilizing interlayer excitons at the TMDC-QW interface. Our observations establish an approach for engineering optical response and exciton dynamics in mixed-dimensional HJs through precise barrier engineering.

## Experimental Section

In_0.21_Ga_0.79_N thin films were epitaxially grown in a horizontal metal–organic chemical vapor deposition (MOCVD) reactor using trimethylindium (TMIn) and trimethylgallium (TMGa) as metalorganic precursors, with ammonia (NH_3_) as the nitrogen source. The bubbler temperatures for TMIn and TMGa were maintained at 25 °C and −14 °C, respectively, with hydrogen (H_2_) gas serving as the carrier gas. A 430-μm-thick c-plane sapphire substrate was thermally cleaned at 1100 °C for 10 min under a reactor pressure of 100 Torr with an H_2_ gas flow rate of 2000 sccm, followed by a 2 min nitridation stage under identical conditions using a 1:1 NH_3_:H_2_ mixture (2000 sccm each). Subsequently, a 100 nm GaN buffer layer was deposited at 500 °C for 10 min, followed by the growth of a 1.35 μm unintentionally doped GaN layer at 1150 °C for 60 min. During GaN growth, TMGa was introduced at 4 sccm while maintaining reactor pressure and H_2_/NH_3_ flow rates. Finally, a 12 nm In_0.21_Ga_0.79_N layer was grown at 760 °C for 1 min under 300 Torr with 2000 sccm N_2_ and 3000 sccm NH_3_, while TMGa and TMIn were supplied at 1.3 and 20 sccm, respectively.

Trilayer MoS_2_ films were synthesized on SiO_2_/Si substrate using a single-step chemical vapor deposition (CVD) process. A liquid metal precursor was prepared by dissolving 0.8 g of sodium molybdate dihydrate (Na_2_MoO_4_·2H_2_O) in deionized water and isopropyl alcohol at a volumetric ratio of 0.75:0.5:0.1. The precursor was spin-coated onto O_2_ plasma-treated SiO_2_/Si substrate at 3000 rpm for 1 min. The coated substrate was then loaded into a single-zone CVD furnace under ambient atmosphere, where the temperature was ramped up to 750 °C over 7 min, maintained at that temperature for 10 min to promote sulfurization, and allowed to cool naturally to room temperature. The as-grown MoS_2_ layers were subsequently transferred onto Al_2_O_3_/InGaN/GaN, InGaN/GaN, and sapphire substrates by a PMMA-assisted wet transfer method, followed by annealing in a vacuum oven at 90 °C for 5 min to complete film integration.^32^

## Results and Discussion

Spatial separation of charge carriers in III–N structures and 2D material layers enables interlayer excitons with extended lifetimes compared to intralayer excitons. Development of mixed-dimensional HJs offers opportunities to exploit complementary material properties through engineered band alignment for precise interlayer exciton control. Here, we investigated interlayer exciton dynamics in mixed-dimensional HJs combining an InGaN quantum well with MoS_2_ layers through Al_2_O_3_ spacer. Figure 1a illustrates the schematic of the fabricated trilayer MoS_2_/Al_2_O_3_/InGaN QW HJ. The stack consists of an undoped GaN buffer, atop which a single InGaN QW is grown and capped by a 2 nm Al_2_O_3_ barrier layer. A CVD-grown trilayer MoS_2_ is subsequently transferred to complete the junction. Upon illumination with photon energies exceeding the bandgaps, tightly bound Wannier excitons are generated within the MoS_2_,^12,33^ while Wannier-Mott excitons and free carriers are generated in the InGaN QW.^34^ In this configuration, the Al_2_O_3_ spacer spatially confines photogenerated carriers within the InGaN QW and MoS_2_ layers. The photogenerated holes in the MoS_2_ valence band bind with electrons in the InGaN QW across the Al_2_O_3_ barrier, leading to the formation of interlayer excitons (top panel of Figure 1b). The ultrathin Al_2_O_3_ spacer enables close proximity between the MoS_2_ and InGaN layers, allowing strong interlayer Coulomb interactions and thereby facilitating the formation of interlayer excitons.^35–39^ In the absence of the Al_2_O_3_ spacer, direct contact between the MoS_2_ and InGaN layers induces significant PL quenching through interfacial defect states (bottom panel of Figure 1b).

**Figure 1:**
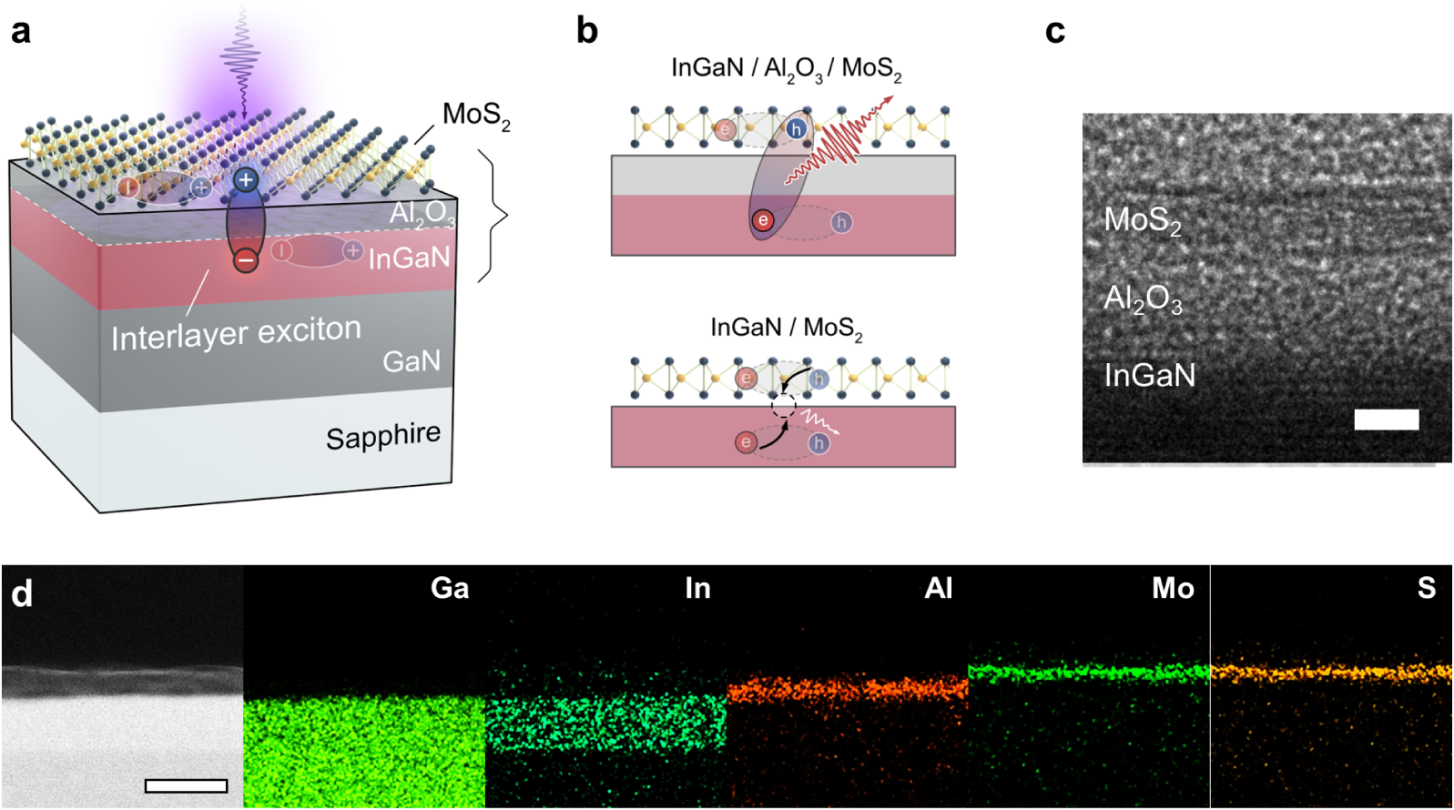
Structural and compositional characterization of MoS_2_/Al_2_O_3_/InGaN quantum well (QW) heterojunctions (HJs). (**a**) Schematic of the MoS_2_/Al_2_O_3_/InGaN QW HJ illustrating the formation of interlayer excitons under light illumination above bandgap. (**b**) Schematic comparison of carrier dynamics in MoS_2_/InGaN HJs with (top) and without (bottom) an Al_2_O_3_ spacer. Direct contact between InGaN and MoS_2_ leads to interfacial defect-mediated nonradiative recombination, suppressing the formation of stable excitons, whereas the Al_2_O_3_ spacer enforces spatial confinement of carriers in distinct layers. (**c**) Cross-sectional high-resolution transmission electron microscopy (HRTEM) image of the MoS_2_/Al_2_O_3_/InGaN QW HJ, confirming uniform layer thicknesses of ∼2 nm for both trilayer MoS_2_ and Al_2_O_3_ (scale bar: 2 nm). (**d**) Scanning transmission electron microscopy (STEM) image and corresponding energy dispersive X-ray spectroscopy (EDS) elemental maps for Ga, In, Al, Mo, and S in the corresponding cross section, demonstrating abrupt and well-defined interfaces without intermixing between layers with a 12 nm-thick InGaN layer (scale bar: 20 nm).

Cross-sectional high-resolution transmission electron microscopy (HRTEM) image (Figure 1c), scanning transmission electron microscopy (STEM) image and corresponding energy-dispersive X-ray spectroscopy (EDS) maps (Figure 1d) confirm the atomically sharp interfaces of the stacked MoS_2_/Al_2_O_3_/InGaN QW HJ. Cross-sectional imaging shows the presence of InGaN (∼12 nm) and Al_2_O_3_ (∼2 nm) layers beneath the contiguous trilayer MoS_2_ film (Figure 1c and S1a). Complementary atomic force microscopy (AFM) measurements (Figure S2) further reveal that the RMS roughness decreases from 0.52 to 0.32 nm upon Al_2_O_3_ deposition, confirming that Al_2_O_3_ spacer contributes to improved surface smoothness and is uniformly deposited across the surface. The crystalline quality of InGaN is demonstrated by the high-angle annular dark field (HAADF) STEM image (Figure S1b), which clearly resolves d(0002) lattice fringes, confirming the high crystallinity and the *c*-axis-oriented growth of the film. Consistent with this observation, electron channeling contrast image (ECCI) analysis revealed a threading dislocation density of ∼1.3 × 10^9^ cm^–2^ (Figure S3), a value comparable to previously reported InGaN QWs on sapphire substrate that still maintain reasonable internal quantum efficiency up to 26%.^40,41^ EDS elemental maps for In, Ga, Mo, S, and Al further demonstrate well-defined interfaces without evidence of interdiffusion.

Figure 2a and b depict the band alignment of the trilayer MoS_2_/InGaN QW HJ with and without an Al_2_O_3_ spacer, respectively. The energy levels are determined by first-principles density functional theory (DFT) and corroborated by the emergence of a new PL emission peak. With the Al_2_O_3_ barrier in place, a potential well is formed within the InGaN layer, while its conduction band minimum (CBM) lies 0.22 eV above that of MoS_2_ (Figure 2a). The 2 nm Al_2_O_3_ spacer enables the formation of interlayer excitons that decay radiatively at low temperatures with an emission energy of 2.02 eV. Additionally, the Al_2_O_3_ layer passivates the surface defects in the InGaN layer and suppresses interfacial nonradiative recombination pathways.^42,43^ By contrast, direct contact between MoS_2_ and InGaN in the absence of the Al_2_O_3_ spacer yields interfacial defect states, particularly on the InGaN surface, which facilitate rapid nonradiative recombination and hinder the formation of stable excitons (Figure 2b). The potential barrier induced by Al_2_O_3_ suppresses interfacial annihilation while the sufficiently thin Al_2_O_3_ barrier, less than the Bohr radii of the excitons in MoS_2_ (∼0.5 nm) and InGaN (∼3.4 nm), ensures adequate overlap of electron–hole wave functions, thereby stabilizing interlayer exciton states.^44–46^

**Figure 2:**
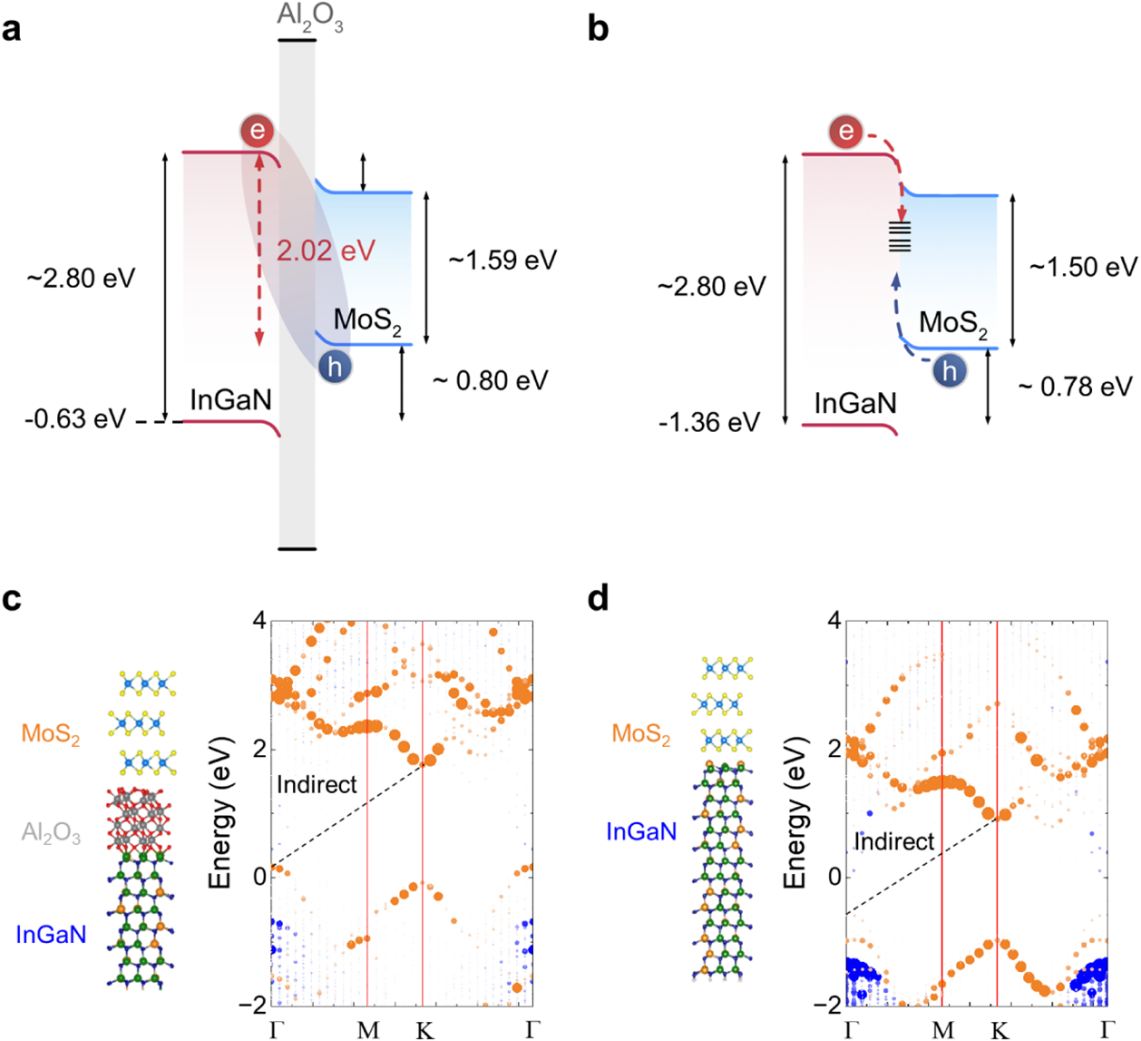
Band alignment and electronic structure analysis of MoS_2_/InGaN quantum well heterojunctions with and without an Al_2_O_3_ spacer. (a) Energy-band diagram of MoS_2_/InGaN QW HJ incorporating a 2 nm Al_2_O_3_ barrier, showing the formation of a confined potential well that spatially separates photoexcited carriers, leading to the formation of interlayer excitons with holes in MoS_2_ and electrons in InGaN. (**b**) Corresponding band diagram for direct MoS_2_–InGaN contact without the Al_2_O_3_ spacer, illustrating nonradiative recombination through interfacial defect states, which suppresses stable exciton formation. (**c**) Atomic model and projected band structures of the individual layers in the MoS_2_/Al_2_O_3_/InGaN QW HJ, calculated using density functional theory (DFT). The contributions from MoS_2_ and InGaN are shown in orange and blue, respectively, revealing a high density of states (DOS) near the valence band maximum (VBM) at the Γ point originating from MoS_2_. (**d**) In the absence of the Al_2_O_3_ spacer, DOS near the VBM of MoS_2_ at the Γ point is reduced, while InGaN exhibits an increased DOS in the 0–2 eV range, associated with surface defect states. Additionally, both MoS_2_ and InGaN bands shift toward lower energy levels upon direct contact, with the MoS_2_ bands exhibiting a slightly reduced indirect bandgap due to strain.

We verified the electronic band structure of the MoS_2_/Al_2_O_3_/InGaN (Figure 2c) and MoS_2_/InGaN (Figure 2d) HJs via DFT calculations. Supercells consisted of trilayer MoS_2_ interfaced with an In_0.21_Ga_0.79_N quantum well, with and without a 2 nm amorphous Al_2_O_3_ spacer. Figure 2c and d display the atomic structures and their projected band dispersions of MoS_2_ (red) and InGaN (blue) layers. Owing to its multilayer nature, the MoS_2_ exhibits an indirect bandgap of 1.5–1.6 eV, depending on the applied strain,^47^ with CBM at K and the valence band maximum (VBM) at Γ. A relatively higher density of states (DOS) near the valence band maximum at the Γ point originating from MoS_2_ is observed in the MoS_2_/Al_2_O_3_/InGaN, indicating the potential for hole localization in MoS_2_ that supports the formation of interlayer excitons. Additionally, without the Al_2_O_3_ spacer, an increase in DOS associated with surface defect states is observed in the 0–2 eV range, indicating enhanced defect-mediated electronic contributions at the interface. Notably, in the MoS_2_/Al_2_O_3_/InGaN structure, the VBM of MoS_2_ appears at approximately 0.17 eV, which is ∼0.80 eV higher than the VBM of InGaN positioned at −0.68 eV. Although the conduction band of InGaN is not clearly resolved in the DFT due to the presence of indium-related defect states, the CBM is estimated to lie around 2.17 eV based on the band edge-emission peak at ∼2.80 eV. Given that the VBM of MoS_2_ lies at approximately 0.17 eV, the energy separation between the hole in MoS_2_ and the electron in InGaN is estimated to be ∼1.95 eV. This value is in close agreement with the interlayer exciton emission energy (2.02 eV) that we designed through the MoS_2_/Al_2_O_3_/InGaN HJ, with minor discrepancies attributed to differences in exciton binding energy and the inherent gap between theoretical and experimental evaluations.

Although DFT analysis suggests a straddling Type-I band alignment, formation of interlayer excitons remains feasible in the MoS_2_/Al_2_O_3_/InGaN HJ. The ultrathin Al_2_O_3_ spacer suppresses direct tunneling while preserving long-range Coulomb interactions, consistent with prior reports of interlayer excitons across thin dielectrics.^48–51^ In addition, local band bending at the interface with upward bending in MoS_2_ (Figure S4) creates electrostatic fields that emulate Type-II-like separation. These results demonstrate that the combined influence of Coulomb coupling and interfacial band bending enables the formation of interlayer excitons in the MoS_2_/Al_2_O_3_/InGaN HJ.

To complement the electronic structure analysis, we employed X-ray photoelectron spectroscopy (XPS). XPS measurement confirms the absence of interfacial chemical bonding, indicating a van der Waals interface at the heterojunction (Figure 3a–d). Core-level spectra of Ga 2p_3/2_ exhibit a characteristic peak at 1116.6 eV for the bare InGaN QW, MoS_2_/Al_2_O_3_/InGaN, and MoS_2_/InGaN HJs, with negligible binding energy shift among three configurations (Figure 3a). The sharp, single Gaussian peak and absence of additional features indicate minimal surface defects such as Ga_2_O_3_ or Ga–O bonds on the InGaN surface.^52^ Similarly, the In 3d_5/2_ and In 3d_3/2_ core-level spectra exhibit peaks at 443.6 and 451.1 eV, respectively, consistent across all samples (Figure 3b).^52^ In the MoS_2_/InGaN sample, however, a weak shoulder peak appears near 444.5 eV, attributed to In–O bonding, possibly resulting from partial oxidation of the InGaN surface due to the absence of surface passivation.^53^ While the Ga and In core-level spectra remain unchanged, the overall peak intensities, particularly for Ga 2p_3/2_ and In 3d_5/2_, exhibit a noticeable reduction following Al_2_O_3_ deposition. This decrease is attributed to the intrinsic instability of the InGaN surface which is susceptible to interfacial degradation during transfer and environmental exposure. Consistent with this, O 1s spectra (Figure S5) of bare InGaN show contributions from both O–In (529.5 eV) and O–Ga (531.0 eV) bonds whereas in the MoS_2_/Al_2_O_3_/InGaN heterojunction only the O–Al component remains, indicating that native oxides are largely removed or reconfigured during TMA exposure. The introduction of the Al_2_O_3_ spacer is therefore not only essential for its dielectric functionality, but also for stabilizing the buried interface and suppressing nonradiative recombination pathways by physically decoupling MoS_2_ from surface trap states in InGaN.

**Figure 3:**
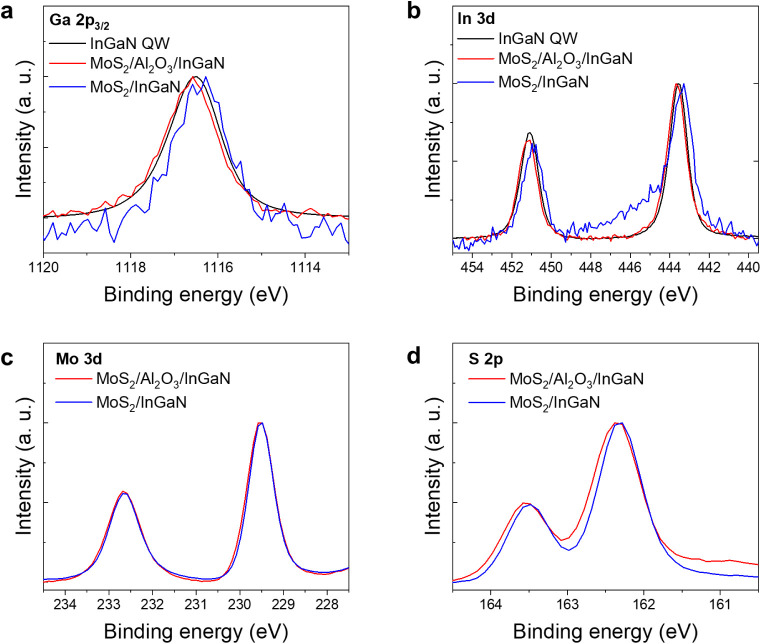
X-ray photoelectron core-level spectra of the InGaN/GaN quantum well and MoS_2_-based heterojunctions. (**a**) Ga 2p_3/2_ spectra for the bare InGaN/GaN QW, MoS_2_/Al_2_O_3_/InGaN HJ, and MoS_2_/InGaN HJ, showing identical binding energies across all configurations. (**b**) In 3d spectra collected from the same three samples, with invariant peak positions indicating unaltered indium chemical states upon heterostructure formation. (**c**) Mo 3d spectra for MoS_2_ in both HJs, demonstrating consistent binding energies and confirming the chemical integrity of MoS_2_. (**d**) S 2p spectra for MoS_2_ in the two junctions. The congruence of binding energies across all core levels indicates negligible chemical interaction or interfacial reaction between MoS_2_ and the InGaN layer in both heterojunction configurations.

Further analysis of the MoS_2_ layer was obtained by examining the Mo 3d (Figure 3c) and S 2p (Figure 3d) core-level spectra. In both the MoS_2_/Al_2_O_3_/InGaN and MoS_2_/InGaN HJs, the Mo 3d spectra exhibit nearly identical sharp peaks at ∼229.5 eV (Mo^4+^ 3d_5/2_) and ∼232.6 eV (Mo^4+^ 3d_3/2_), with narrow full width at half-maximum (FWHM) of 0.6 and 0.7 eV, respectively, indicating high-quality, unoxidized 2H-MoS_2_ phase.^54,55^ The S 2p spectra show the expected doublet for 2H-MoS_2_, with a subtle ∼0.05 eV reduction in FWHM in the MoS_2_/InGaN sample relative to that in MoS_2_/Al_2_O_3_/InGaN. This slight sharpening of the S 2p peak suggests enhanced local bonding uniformity or reduced chemical inhomogeneity at the direct MoS_2_ and InGaN interface, although no binding energy shifts or additional spectral components are observed.

Following the verification of interfacial chemical integrity, we characterized the crystalline quality and In composition of the InGaN quantum well to confirm that the interlayer exciton behavior originates from the designed band alignment rather than from structural defects or compositional variations. To verify the structural integrity of the complete heterostructure, we implemented X-ray diffraction (XRD) analysis. As shown in Figure 4a and b, the diffraction pattern exhibits distinct diffraction peaks at 34.66° and 72.95°, corresponding to the (002) and (004) planes of GaN, respectively, whereas broader peaks at 33.87° and 71.12° are attributed to the (002) and (004) reflections of InGaN. These well-defined peaks, along with the absence of extraneous reflections, confirm epitaxial single-crystalline wurtzite growth for both GaN and InGaN layers. A weak diffraction peak at 14.50° is attributed to the MoS_2_ (002) reflection, validating its integration atop the Al_2_O_3_/InGaN/GaN stack. Additionally, a peak at 41.79° corresponds to the Al_2_O_3_ (006) plane, originating from the underlying c-plane sapphire substrate rather than the amorphous or polycrystalline ALD-deposited Al_2_O_3_ barrier.^56,57^ A magnified view of the GaN (002) and InGaN (002) peaks (Figure 4b) clearly resolves their respective 2θ positions. By applying Bragg’s law, the out-of-plane lattice constants of GaN and InGaN are calculated to be 5.178 Å and 5.294 Å, respectively. To estimate the indium composition, we assumed that the 12 nm InGaN layer is pseudomorphically strained to the underlying GaN layer, which was epitaxially grown on a c-plane sapphire substrate, and applied a simplified Vegard’s law approximation. Based on the measured out-of-plane lattice constants of GaN (5.178 Å) and InGaN (5.294 Å), along with the bulk lattice constant of InN (5.703 Å), the In composition in the quantum well was estimated to be approximately 21% (In_0.21_Ga_0.79_N).^58,59^ A complementary ECCI analysis further confirmed a threading dislocation density (TDD) of ∼1.3 × 10^9^ cm^–2^. Although the TDD is relatively high compared to those grown on AlN buffer films, it did not substantially compromise the PL characteristics or exciton dynamics of the heterostructure.^40,41^

**Figure 4:**
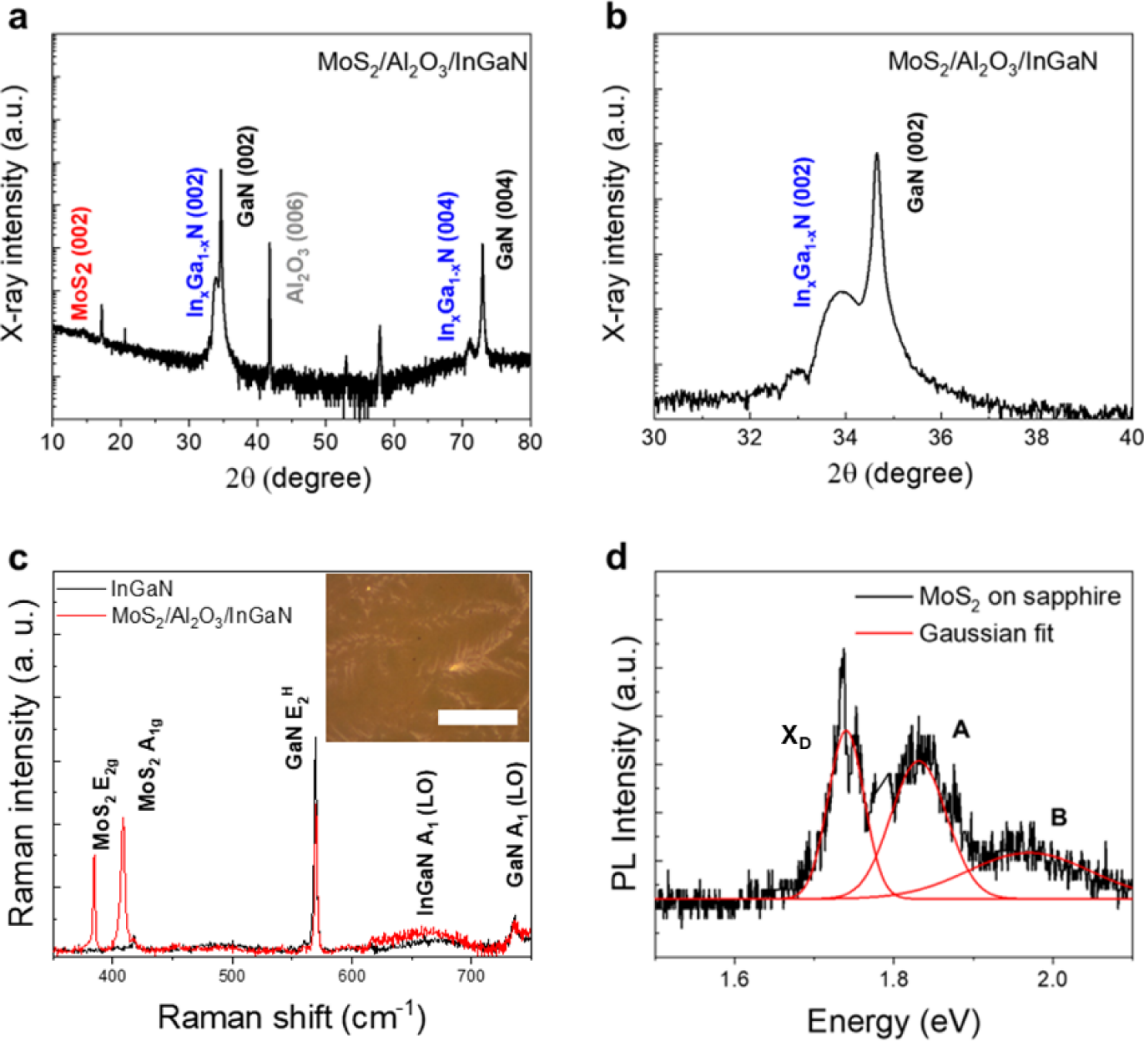
Structural and optical characterization of the MoS_2_/Al_2_O_3_/InGaN heterojunction. (**a**) X-ray diffraction (XRD) pattern of the MoS_2_/Al_2_O_3_/InGaN heterojunction (HJ), showing diffraction peaks at 34.66° and 72.95° corresponding to the (002) and (004) planes of wurtzite GaN, and at 33.87° and 71.12° corresponding to the (002) and (004) planes of InGaN. A weak peak at 14.50° is attributed to the (002) reflection of trilayer MoS_2_, while the peak at 41.79° arises from the (006) plane of the sapphire substrate. (**b**) Expanded view of the InGaN (002) and GaN (002) diffraction peaks, illustrating the slight lattice mismatch between the two nitrides. (**c**) Raman spectra of the MoS_2_/Al_2_O_3_/InGaN HJ, displaying characteristic peaks of trilayer MoS_2_ at 384.5 cm^–1^ and 408.4 cm^–1^, corresponding to the in-plane E^1^_2g_ and out-of-plane A_1g_ vibrational modes, respectively. Peaks at 569.5 cm^–1^ and 736.0 cm^–1^ are assigned to the E_2_^H^ and A_1_(LO) phonon modes of GaN. A peak near 690 cm^–1^ corresponds to the A_1_(LO) mode of InGaN. The inset shows an optical microscopic image of the MoS_2_/Al_2_O_3_/InGaN HJ with scale bar of 50 μm. (**d**) Room-temperature confocal photoluminescence spectrum of trilayer MoS_2_ on a sapphire substrate. Gaussian deconvolution resolves three distinct emission peaks at 1.74, 1.85, and 1.97 eV, corresponding to the defect-bound exciton (X_D_), the A exciton, and the B exciton transitions, respectively.

To ensure accurate layer identification and consistent characterization, the transferred MoS_2_ flakes were initially examined using optical microscopy (inset of Figure 4c). Regions with brighter contrast correspond to locally thicker multilayer domains whereas the majority of the flake exhibits darker contrast consistent with trilayer thickness. This was further verified by Raman spectroscopy and confocal PL analyses. The phonon spectra and vibrational dynamics of the MoS_2_/Al_2_O_3_/InGaN HJ were investigated through Raman spectroscopic measurements (Figure 4c). In the bare InGaN quantum well, Raman peaks at 569.5 cm^–1^ and 736.0 cm^–1^, corresponding to the high frequency E_2_^H^ and longitudinal optical A_1_(LO) modes of GaN, respectively, are observed and remain unaltered upon integration with MoS_2_. In the heterojunction, two additional peaks at 384.5 cm^–1^ and 408.4 cm^–1^ correspond to the E^1^_2g_ and A_1g_ modes of MoS_2_, which arise from the in-plane vibrations of Mo and S atoms and the out-of-plane vibrations of S atoms, respectively. The separation between these two modes is commonly used to determine the layer number of the MoS_2_, as the A_1g_ mode shifts with increasing thickness and the E^1^_2g_ mode is influenced by interlayer coupling.^59,60^ The measured mode separation of 23.9 cm^–1^ therefore indicates the presence of trilayer MoS_2_.^61^ In addition to layer identification, the Raman peak positions and line widths provide direct evidence of strain in the MoS_2_ layer (Figure S6). When MoS_2_ is placed directly on InGaN without the Al_2_O_3_ spacer, the E_2g_ mode redshifts from ∼384.5 to ∼383.2 cm^–1^ accompanied by a line width broadening from 1.8 to 3.8 cm^–1^, while the A_1g_ mode remains nearly unchanged. This behavior indicates the presence of in-plane tensile strain which reduces the bandgap of MoS_2_. The origin of this strain is potentially attributed to the surface roughness of bare InGaN, as shown by AFM measurements (Figure S2).

To further establish the intrinsic photoluminescence characteristics of the trilayer MoS_2_ and confirm its layer thickness independent of the underlying quantum well structure, a trilayer MoS_2_ film on a sapphire substrate was characterized by confocal PL spectroscopy. The resulting spectrum exhibits three prominent peaks at approximately 1.74, 1.85, and 1.97 eV, corresponding to the defect-bound exciton (X_D_), the A exciton, and the B exciton transitions of MoS_2_, respectively (Figure 4d).^62–64^ Compared with monolayer MoS_2_, the A exciton peak on the trilayer sample is red-shifted and broadened with reduced PL intensity. These changes are attributed to interlayer coupling and enhanced dielectric screening which reduce the exciton binding energy. The observed energy separation between A and B excitons, approximately 120 meV, aligns with reported values for trilayer MoS_2_.^63^ These PL results, in agreement with the Raman analysis, confirm that the transferred MoS_2_ film is composed of three atomic layers.

Having established the baseline MoS_2_ properties, we investigated the formation of interlayer excitons in the complete heterojunction structure through low-temperature PL measurements. Low-temperature PL spectra were acquired in a He-cryostat under 325 nm He–Cd laser excitation to confirm the formation of interlayer excitons in the MoS_2_/Al_2_O_3_/InGaN HJ. At 17 K, the InGaN QW exhibits a sharp band-edge emission at 2.80 eV and a broad peak near 2.32 eV corresponding to the defect states in the GaN (Figure 5a).^19,65,66^ Under these conditions, PL emission from the multilayer MoS_2_ film is barely observed, due to the relatively thin optical cross section of the MoS_2_ compared with the InGaN/GaN film.

**Figure 5:**
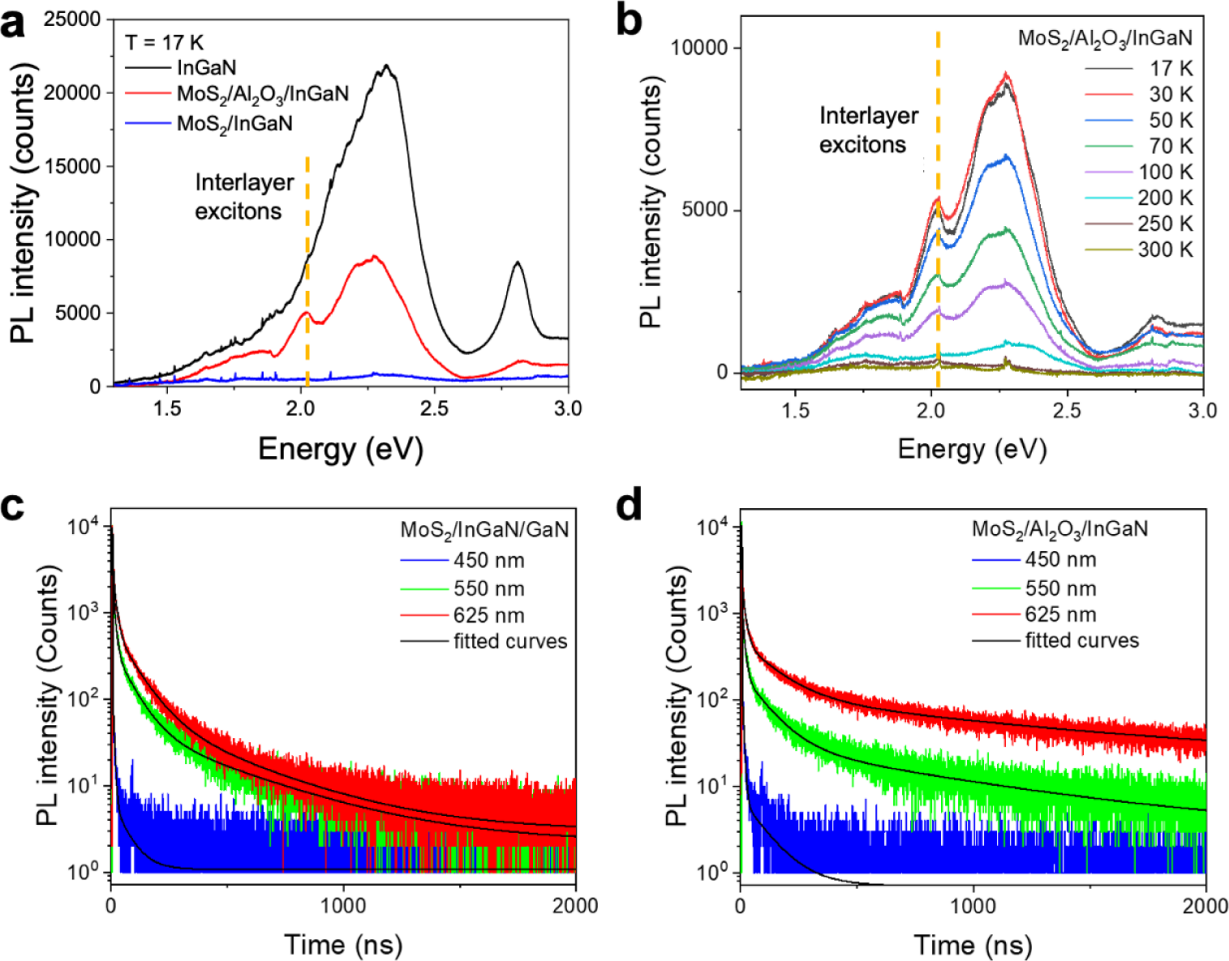
Optical analysis of InGaN/GaN quantum well and MoS_2_-based heterojunctions. (**a**) Low-temperature (17 K) photoluminescence (PL) spectra of the bare InGaN/GaN QW (black), MoS_2_/Al_2_O_3_/InGaN HJ (red), and MoS_2_/InGaN HJ (blue). A distinct emission peak at ∼2.02 eV appears exclusively in the MoS_2_/Al_2_O_3_/InGaN structure, consistent with radiative recombination of interlayer excitons. (**b**) Temperature-dependent PL spectra (17–300 K) of the MoS_2_/Al_2_O_3_/InGaN HJ, showing that the interlayer exciton-related emission persists up to 100 K without any spectral shift, and is fully quenched above 200 K. (**c**) Time-resolved PL (TR-PL) decay curves of the MoS_2_/InGaN HJ, measured with 450 nm (blue), 550 nm (green), and 625 ± 25 nm (red) bandpass filters. The 450 nm signal exhibits fast decay dynamics, with a dominant lifetime of approximately 0.33 ns, characteristic of band-edge emission from InGaN. In contrast, the 550 and 625 nm signals show extended decay with lifetimes up to ∼438 ns, associated with defect-related states in GaN. (**d**) TR-PL decay curves for the MoS_2_/Al_2_O_3_/InGaN HJ, highlighting the extended lifetime of the 625 nm signal up to 1.12 μs attributed to the presence of interlayer excitons.

On the other hand, when MoS_2_ is in direct contact with InGaN QW without the Al_2_O_3_ spacer, the InGaN band-edge emission is completely quenched, indicating that the Al_2_O_3_ barrier plays a crucial role in preventing nonradiative recombination at intrinsic surface defects of the InGaN QW.^67–70^ This quenching represents a critical challenge for interlayer exciton formation, as it prevents radiative recombination across the heterointerface. Notably, introduction of the Al_2_O_3_ barrier suppresses the quenching of electron–hole pairs and gives rise to a distinct additional emission at 2.02 eV. This new spectral feature is ascribed to radiative recombination of interlayer excitons formed across the MoS_2_/Al_2_O_3_/InGaN junction. Simultaneously, the original 2.80 eV InGaN band-edge peak is significantly weakened, indicating that excitons generated in the InGaN QW are predominantly diverted from intralayer recombination toward formation of excitons across the MoS_2_/Al_2_O_3_/InGaN junction. Confocal PL measurements further support this behavior by exhibiting a reduction in the MoS_2_ excitonic emission upon formation of MoS_2_/Al_2_O_3_/InGaN HJ (Figure S7).

Temperature-dependent PL measurements of the MoS_2_/Al_2_O_3_/InGaN HJ reveal that, up to 50 K, the interlayer exciton peak remains well-resolved with high intensity and a narrow line width, indicative of stable exciton coupling (Figure 5b). As the temperature increases, the interlayer exciton emission gradually quenches without any observable energy shift, attributed to thermally activated dissociation of excitons and enhanced phonon scattering.^71^ The lack of a temperature-dependent spectral shift confirms that the emission does not originate from the B exciton transition in MoS_2_, which typically redshifts with increasing temperature.^72^ Additionally, the suppression of the InGaN band-edge emission further implies increased exciton–phonon interactions. At temperatures exceeding 200 K, thermal energy compromises the stability of interlayer excitons, leading to a significant reduction in interlayer excitonic emissions. Although the interlayer exciton becomes indistinct in micro-PL measurements above ∼200 K due to spatial averaging across nonuniform MoS_2_ regions, TRPL analysis reveal that a weak excitonic emission near 2.02 eV with prolonged lifetimes persists even at room temperature, albeit contributing only modestly to the overall emission.

Time-resolved PL (TR-PL) measurements were performed to probe carrier dynamics in the MoS_2_/InGaN (Figure 5c) and MoS_2_/Al_2_O_3_/InGaN (Figure 5d) HJs. Spatially localized excitation was achieved via a confocal microscope, with a single-mode pulsed diode laser (375 nm with 30 ps pulse width and average power of 1–4 μW operating in 200 kHz repetition rate) employed as the excitation source. Emitted photons were collected through a dichroic mirror and long-pass filter, passed through a 150 μm pinhole, and directed by mirrors into the detection path. Bandpass filters (450 ± 20, 550 ± 20, and 625 ± 25) were placed in front of the avalanche photodiode detector to selectively isolate emission within distinct spectral windows. This configuration enabled selective temporal resolution of PL from the heterojunction region.

In the MoS_2_/InGaN heterostructure (Figure 5c), the PL decay curves at 450, 550, and 625 nm exhibit multiexponential behavior, indicating the coexistence of distinct recombination pathways. The decay profiles were fitted with a biexponential model at 450 nm and with a triexponential model at 550 and 625 nm for both MoS_2_/InGaN and MoS_2_/Al_2_O_3_/InGaN HJ. Notably, for the MoS_2_/Al_2_O_3_/InGaN structure at 625 nm, an additional long-lived component was required to capture the decay profile which we attribute to interlayer excitons recombination. The extracted decay constants (τ_1_, τ_2_, τ_3_, and τ_4_) with their corresponding amplitudes for both HJs are provided in Supporting Information Tables S1 and S2, respectively. The emission at 450 nm, attributed to band-edge recombination in InGaN, decays with a dominant lifetime of approximately 0.33 ns, in agreement with literature values for InGaN QWs.^73,74^ A slower component (4.45 ns) originates from recombination assisted by localized defect states. In contrast, emissions at 550 and 625 nm, primarily associated with deep-level defect states in GaN, exhibit substantially longer lifetimes up to 95 ns.^65^ Upon introduction of the Al_2_O_3_ spacer (Figure 5d), the defect-associated lifetimes (4.45 and 95 ns) remain observable, indicating that defect-mediated recombination channels persist in the heterostructure. At the same time, the PL lifetimes exhibit a noticeable increase, particularly at 625 nm where the decay extends to approximately 1.12 μs, confirming suppressed nonradiative decay and enhanced carrier relaxation dynamics at the passivated interface. This prolonged lifetime indicates the formation of interlayer excitons.^75–78^ The Al_2_O_3_ barrier serves to spatially confine charge carriers within the MoS_2_ and InGaN layers, thereby enhancing their radiative lifetimes and facilitating interlayer exciton coupling across the heterojunction.

## Conclusions

In summary, we have engineered a mixed-dimensional heterojunction comprising trilayer MoS_2_ and an InGaN QW separated by an Al_2_O_3_ barrier, thereby enabling the formation of interlayer excitons. Controlled barrier thickness yields a characteristic 2.02 eV emission, attributed to radiative interlayer exciton recombination. The suppression of the InGaN band-edge emission demonstrates that carriers generated within the QW preferentially couple to the carriers in the MoS_2_ layer. Nevertheless, temperature-dependent PL analysis provides clear evidence of interlayer excitons remaining stable up to ∼100 K, with quenching above 200 K due to enhanced exciton–phonon interactions and thermal dissociation. These results establish that a sub-Bohr-radius dielectric barrier can both stabilize interlayer exciton states and mitigate nonradiative losses, offering a versatile strategy for modulating exciton dynamics in mixed-dimensional heterojunctions and providing a foundation for the development of optoelectronic devices based on interlayer excitonic dynamics.

## Supplementary Material



## Data Availability

The data related to the figures and other findings of this study are available from the corresponding author upon reasonable request.

## Notes

Cross-sectional HAADF STEM images of the MoS_2_/Al_2_O_3_/InGaN QW HJ; AFM images of the InGaN QW surface before and after Al_2_O_3_ deposition; ECCI of the InGaN QW surface; spatially resolved projected band structure of the MoS_2_/Al_2_O_3_/InGaN HJ; XPS O 1s spectra of the InGaN QW and MoS_2_/Al_2_O_3_/InGaN QW HJ; Raman spectra of the InGaN/GaN QW and MoS_2_-based HJs; confocal PL spectra of bare InGaN QW and MoS_2_/Al_2_O_3_/InGaN QW HJ at room temperature; summary of multiexponential fits to the TR-PL decay profiles at 450, 550, and 625 nm for MoS_2_-based HJs (PDF)
