# Supplementary material for: Observation of Interlayer Excitons in Mixed-Dimensional MoS2 and InGaN/GaN Quantum Well Heterojunctions
Source: ACS Appl Mater Interfaces. 2025 Nov 18;17(50):68265–75. doi: 10.1021/acsami.5c16066 (PMC12723632; doi:10.1021/acsami.5c16066)
Supplement: Supplementary file 1 [file am5c16066_si_001.pdf]

Supporting Information

Observation of Interlayer Excitons in Mixed Dimensional MoS_2 and InGaN/GaN Quantum Well Heterojunctions

Do Wan Kim,[1†] Seokje Lee,[2,3†], Yongmin Baek,[1,4†] Kangmin Jeon,[5] Jinwoo Song,[5] Kwangsik Jeong,[6] Seungjin Lee,[7,8] Jae Woo Kim,[7,8] Seokho Kim,[1] Gi Wan Jeon,[9] Ki Kang Kim,[7,8,10] Gyu-Chul Yi,[2] Dong Hyuk Park,[5] Kyusang Lee[1,11*]*

[1]Department of Electrical and Computer Engineering, University of Virginia, Charlottesville, VA 22904, United States

[2]Department of Physics and Astronomy, Institute of Applied Physics, Seoul National University, Seoul, 08826, Republic of Korea

[3]Research Laboratory of Electronics, Massachusetts Institute of Technology, Cambridge, MA 02139, United States

[4]Department of Mechanical Engineering, Seoul National University, Seoul, 08826, Republic of Korea

[5]Department of Chemical Engineering, Program in Biomedical Science and Engineering, Inha University, Incheon 22212, Republic of Korea

[6]Department of Physics, Yonsei University, Gangwon-do 26493, Republic of Korea

[7]Department of Energy Science, Sungkyunkwan University (SKKU), Suwon, 16419, Republic of Korea

[8]Center for Integrated Nanostructure Physics (CINAP), Institute for Basic Science (IBS), Sungkyunkwan University (SKKU), Suwon, 16419, Republic of Korea

[9]Particle Beam Research Division, Korea Atomic Energy Research Institute (KAERI), Gyeongju-Si, Gyeonbuk 38180, Republic of Korea

[10]Department of Physics, Sungkyunkwan University (SKKU), Suwon, 16419, Republic of Korea

[11]Department of Materials Science and Engineering, University of Virginia, Charlottesville, VA 22904, United States

* E-mail: kl6ut@virginia.edu

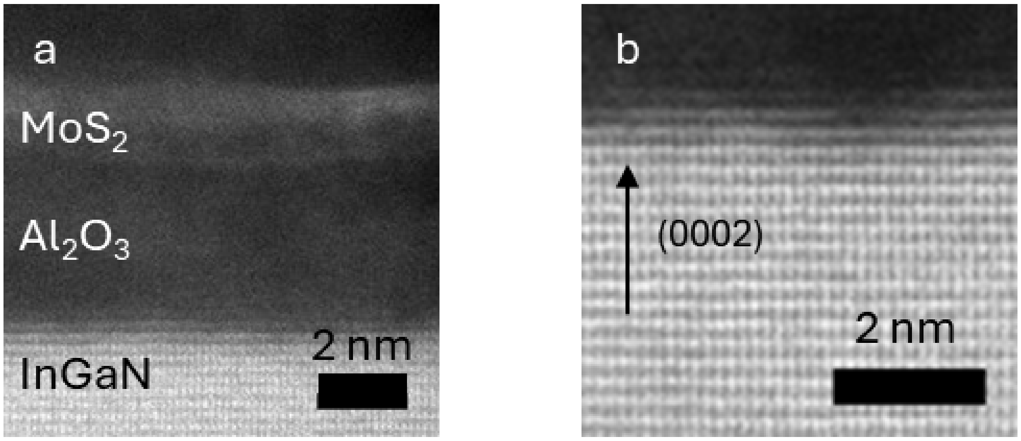

Figure S1. Cross-sectional high-angle annular dark field (HAADF) scanning transmission electron microscopy (STEM) images of MoS_2/Al_2O_3/InGaN quantum well heterojunction in different regions. a. The Al_2O_3 spacer appears ~2.5 nm thick showing a slight fluctuation in the apparent thickness arising from local roughness and focus limitations. b. A magnified image focused on the InGaN region, where the (0002) lattice fringes are clearly resolved, confirming the high crystalline quality and c-axis-oriented growth of InGaN.

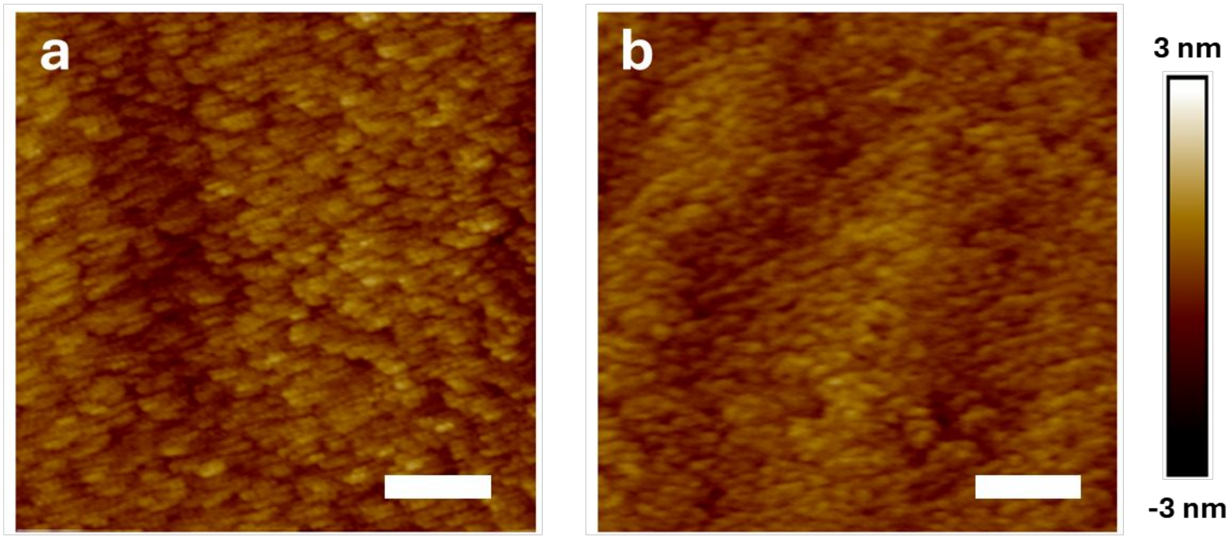

Figure S2. Surface morphology of InGaN quantum well. a. Atomic force microscopy (AFM) image of the InGaN quantum well surface. The root mean square (RMS) roughness is calculated to be 0.52 nm with grain size of ~50 nm. b. After deposition of Al_2O_3, RMS roughness slightly decreased to 0.32 nm with grain size of ~90 nm, indicating that the Al_2O_3 layer smooths the interface and contributes to strain relaxation in the overlying MoS_2.[1]

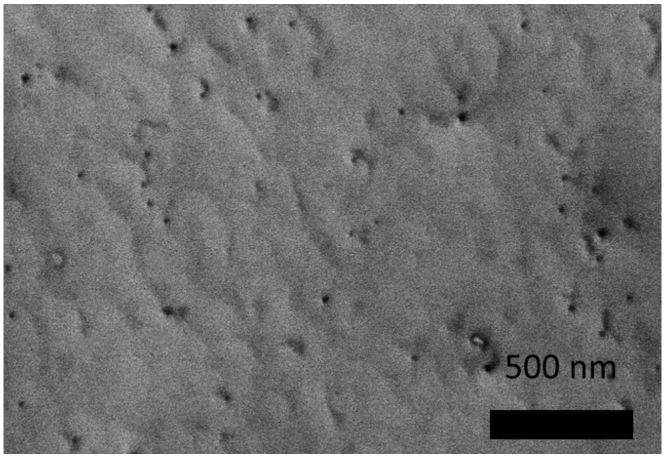

Figure S3. Electron channeling contrast image (ECCI) of the InGaN quantum well surface used for threading dislocation density (TDD) analysis. Dark contrast spots correspond to threading dislocations, from which the TDD was estimated to be ~1.3×10^9 cm^{-2}.

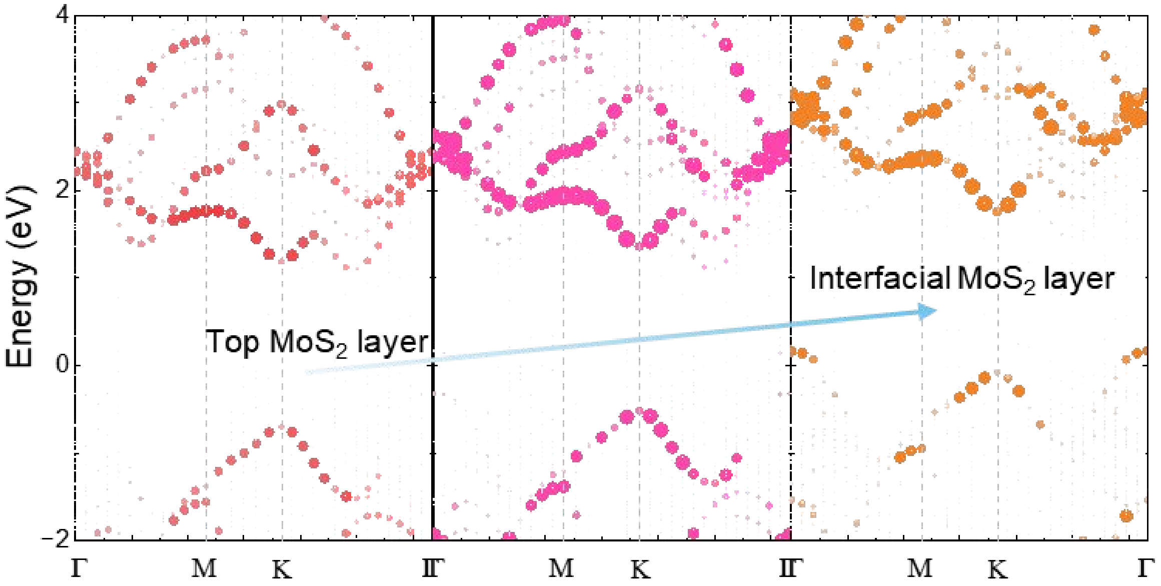

Figure S4. Local band bending of MoS_2 at the interface. Projected band structure of the MoS_2/Al_2O_3/InGaN heterojunction obtained from DFT calculations, with the contributions from individual MoS_2 layers resolved separately. The top (red), middle (pink), and bottom (orange) layers of the MoS_2 are shown, where the bottom layer corresponds to the interfacial layer directly contacting Al_2O_3. An upward band bending is observed toward the interface, with the valence band maximum shifting progressively higher from the top to the bottom MoS_2 layer, thereby providing a favorable driving force for hole accumulation in the interfacial MoS_2 layer.

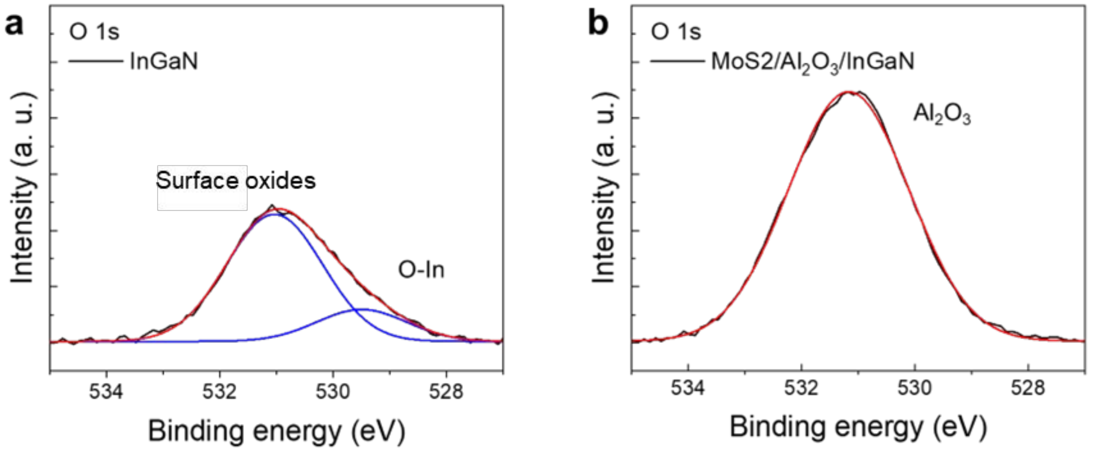

Figure S5. Encapsulation of InGaN surface with Al_2O_3. a. X-ray photoelectron (XPS) O 1s spectrum of the bare InGaN quantum well (QW). The O 1s spectrum is deconvoluted into two Gaussian components at binding energies of 529.5 eV and 531.0 eV, corresponding to O–In bonding and oxygen associated with non-stoichiometric surface oxides,[2,3] respectively. b. XPS O 1s spectrum of MoS_2/Al_2O_3/InGaN QW heterojunction, exhibiting only O-Al peak (531.0 eV) due to partial removal and reconfiguration of native oxide species during TMA exposure.

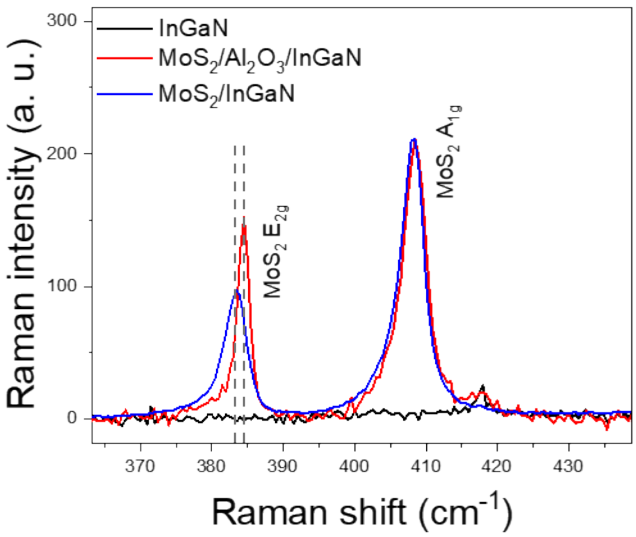

Figure S6. Raman spectra of the InGaN/GaN quantum well and MoS_2-based heterojunctions. The E_{2g} mode exhibits a redshift from ~384.5 to ~383.2 cm^{-1} with significant linewidth broadening when MoS_2 is placed directly on InGaN, whereas the A_{1g} mode remains nearly unchanged. This selective shift indicates the presence of in-plane tensile strain in the direct MoS_2/InGaN interface, which is alleviated by the insertion of the Al_2O_3 spacer. The strain level is estimated to be ~0.2%, sufficient to perturb the electronic structure while not splitting the E'^{+} and E'^{-} modes.[4–6]

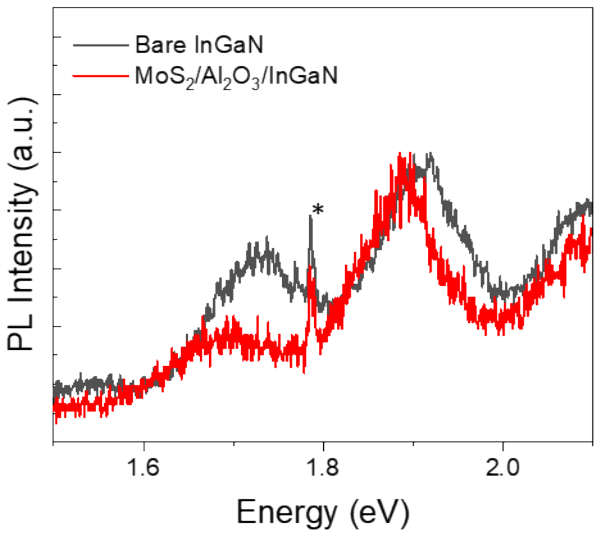

Figure S7. Confocal photoluminescence (PL) spectra of bare InGaN quantum well (QW) and MoS_2/Al_2O_3/InGaN QW heterojunction (HJ) at room temperature. In both samples, the PL spectra are dominated by defect-related emission from InGaN, accompanied by Fabry–Perot interference features. The intrinsic MoS_2 emission is strongly suppressed in the MoS_2/Al_2O_3/InGaN HJ, consistent with the formation of interlayer excitons. The asterisks denote emissions originating from the sapphire substrate.[7,8]

Table S1. Summary of multi-exponential fits to the time-resolved photoluminescence decay profiles at 450, 550, and 625 nm for MoS_2/InGaN heterojunction. The 450 nm data were well described by a bi-exponential model, while the 550 nm and 625 nm datasets required three-exponential fits to capture the recombination dynamics. The extracted decay constants and their corresponding amplitudes are listed.

MoS_2/InGaN	*450 nm*	*550 nm*	*625 nm*
A_1 (counts)	1400 ± 540	7800 ± 250	6800 ± 290
τ_1 (s)	0.33 ± 0.01	0.89 ± 0.08	1.57 ± 0.01
A_2 (counts)	135 ± 8	2100 ± 150	2500 ± 120
τ_2 (s)	4.45 ± 0.08	8.9 ± 0.55	14 ± 0.90
A_3 (counts)	-	359 ± 18	550 ± 23
τ_3 (s)	-	71 ± 3	95 ± 3.40

Table S2. Summary of multi-exponential fits to the time-resolved photoluminescence decay profiles at 450, 550, and 625 nm for MoS_2/Al_2O_3/InGaN heterojunction. The 450 nm data were well described by a bi-exponential model, while the 550 nm and 625 nm datasets required three-exponential fits to capture the recombination dynamics. The extracted decay constants and their corresponding amplitudes are listed.

MoS_2/Al_2O_3/InGaN	*450 nm*	*550 nm*	*625 nm*
A_1 *(counts)*	13000 ± 910	13000 ± 430	6700 ± 260
τ_1 *(s)*	0.35 ± 0.02	0.42 ± 0.03	0.97 ± 0.00
A_2 *(counts)*	157 ± 8	1040 ± 45	1650 ± 37
τ_2 *(s)*	5.7 ± 0.41	9.4 ± 0.60	14 ± 0.14
A_3 *(counts)*	-	173 ± 6	364 ± 3
τ_3 *(s)*	-	99.6 ± 2.7	134 ± 3.30
A_4 *(counts)*	-	-	94 ± 1
τ_4 *(s)*	-	-	1122 ± 16

References

(1) Myers, T. J.; Throckmorton, J. A.; Borrelli, R. A.; O'Sullivan, M.; Hatwar, T.; George, S. M. Smoothing Surface Roughness Using Al2O3 Atomic Layer Deposition. *Appl. Surf. Sci.* **2021**, *569*, 150878. https://doi.org/10.1016/j.apsusc.2021.150878.

(2) Chuang, T. J.; Brundle, C. R.; Rice, D. W. Interpretation of the X-Ray Photoemission Spectra of Cobalt Oxides and Cobalt Oxide Surfaces. *Surf. Sci.* **1976**, *59* (2), 413–429. https://doi.org/10.1016/0039-6028(76)90026-1.

(3) Yang, Y.-G.; Ma, H.-L.; Xue, C.-S.; Zhuang, H.-Z.; Hao, X.-T.; Ma, J.; Teng, S.-Y. Preparation and Structural Properties for GaN Films Grown on Si (1 1 1) by Annealing. *Appl. Surf. Sci.* **2002**, *193* (1), 254–260. https://doi.org/10.1016/S0169-4332(02)00490-7.

(4) Conley, H. J.; Wang, B.; Ziegler, J. I.; Haglund, R. F. Jr.; Pantelides, S. T.; Bolotin, K. I. Bandgap Engineering of Strained Monolayer and Bilayer MoS2. *Nano Lett.* **2013**, *13* (8), 3626–3630. https://doi.org/10.1021/nl4014748.

(5) J. W. Christopher; M. Vutukuru; D. Lloyd; J. S. Bunch; B. B. Goldberg; D. J. Bishop; A. K. Swan. Monolayer MoS2 Strained to 1.3% With a Microelectromechanical System. *J. Microelectromechanical Syst.* **2019**, *28* (2), 254–263. https://doi.org/10.1109/JMEMS.2018.2877983.

(6) Datye, I. M.; Daus, A.; Grady, R. W.; Brenner, K.; Vaziri, S.; Pop, E. Strain-Enhanced Mobility of Monolayer MoS2. *Nano Lett.* **2022**, *22* (20), 8052–8059. https://doi.org/10.1021/acs.nanolett.2c01707.

(7) Reuter, E. E.; Zhang, R.; Kuech, T. F.; Bishop, S. G. Photoluminescence Excitation Spectroscopy of Carbon-Doped Gallium Nitride. *MRS Internet J. Nitride Semicond. Res.* **1999**, *4* (1), 363–368. https://doi.org/10.1557/S1092578300002738.

(8) Huang, C.-C.; Al-Saab, F.; Wang, Y.; Ou, J.-Y.; Walker, J. C.; Wang, S.; Gholipour, B.; Simpson, R. E.; Hewak, D. W. Scalable High-Mobility MoS2 Thin Films Fabricated by an Atmospheric Pressure Chemical Vapor Deposition Process at Ambient Temperature. *Nanoscale* **2014**, *6* (21), 12792–12797. https://doi.org/10.1039/C4NR04228J.
